# Identification and Genomic Analysis of a Novel Group C Orthobunyavirus Isolated from a Mosquito Captured near Iquitos, Peru

**DOI:** 10.1371/journal.pntd.0004440

**Published:** 2016-04-13

**Authors:** Todd J. Treangen, George Schoeler, Adam M. Phillippy, Nicholas H. Bergman, Michael J. Turell

**Affiliations:** 1 National Biodefense Analysis and Countermeasures Center, Frederick, Maryland, United States of America; 2 Department of Entomology, U. S. Naval Medical Research Unit No. 6, Callao, Peru; 3 Virology Division, United States Army Medical Research Institute of Infectious Diseases, Fort Detrick, Maryland, United States of America; Centers for Disease Control and Prevention, UNITED STATES

## Abstract

Group C orthobunyaviruses are single-stranded RNA viruses found in both South and North America. Until very recently, and despite their status as important vector-borne human pathogens, no Group C whole genome sequences containing all three segments were available in public databases. Here we report a Group C orthobunyavirus, named El Huayo virus, isolated from a pool of *Culex portesi* mosquitoes captured near Iquitos, Peru. Although initial metagenomic analysis yielded only a handful of reads belonging to the genus *Orthobunyavirus*, single contig assemblies were generated for L, M, and S segments totaling over 200,000 reads (~0.5% of sample). Given the moderately high viremia in hamsters (>10^7^ plaque-forming units/ml) and the propensity for *Cx*. *portesi* to feed on rodents, it is possible that El Huayo virus is maintained in nature in a *Culex portesi*/rodent cycle. El Huayo virus was found to be most similar to Peruvian Caraparu virus isolates and constitutes a novel subclade within Group C.

## Author Summary

Arthropod-borne viruses remain a significant cause of human and domestic animal disease and new viruses are constantly being discovered. RNA virus discovery and assembly remains a challenge due to highly polymorphic genomes, current lack of breadth and depth of publicly available viral genomes, and confounding factors due to host sequence and sequencing biases. We describe the discovery and genome assembly of El Huayo virus, a group C orthobunyavirus isolated from a pool of *Culex portesi* mosquitoes captured near Iquitos, Peru, and named for the Jardin Botanicao Arboretum El Huayo near where the *Cx*. *portesi* from which the virus was isolated were captured. Although orthobunyaviruses are not commonly associated with human disease, Group C members are widespread in Central and South America and known to cause febrile illness. The discovery, and genome assembly, of El Huayo virus may help to explain numerous dengue-like illnesses where *Aedes aegypti* are not commonly found.

## Introduction

The *Orthobunyavirus* genus comprises a diverse set of viral species, represented by multiple serogroups, including: Bunyamwera, California, Group C, and Simbu [1]. Their RNA genome includes three segments (Small [S], Medium [M], and Large [L]). The L segment encodes a RNA polymerase (RdRP); the M segment encodes two glycoproteins (Gc and Gn) in addition to a non-structural protein (NS); and the S segment encodes both a nucleocapsid protein (NP or N protein) and a non-structural protein (NSs) [2, 3]. Group C viruses were first identified in Brazil around 1950. Members of the California serogroup, including La Crosse, California encephalitis, Inkoo, and Tahyna viruses, are known to cause disease in humans [4–8]. Similarly, members of the Bunyamwera serogroup, including Cache Valley and Bunyamwera viruses [9, 10], Simbu serogroup, including Akabane, Iquitos, and Schmallenberg viruses [11–13], and Group C, including Caraparu, Itaya, Marituba, and Oriboca viruses [14–16], are known to cause disease in humans or domestic animals. Because infection with Group C viruses results in a non-differentiated febrile (dengue-like) illness and the lack of available diagnostic assays for these viruses, it has been difficult to associate these viruses with human disease. However, a study by Forshey et al. [17] identified 30 cases of human illness associated with Group C orthobunyaviruses, many of them Caraparu-like, and estimated that about 2.5% of febrile illnesses in the region were due to infection with an orthobunyavirus. The goal of our study was to sample, sequence and assemble a novel member of the genus *Orthobunyavirus* that had been isolated from a pool of *Culex portesi* mosquitoes captured in Peru in order to provide further genomic insights of this potentially disease-causing virus.

## Materials and Methods

### Ethics statement

The animal work was approved by the USAMRIID Institutional Animal Care and Use Committee. Research was conducted under an IACUC approved protocol in compliance with the Animal Welfare Act, PHS Policy, and other Federal statutes and regulations relating to animals and experiments involving animals. The facility where this research was conducted is accredited by the Association for Assessment and Accreditation of Laboratory Animal Care, International and adheres to principles stated in the Guide for the Care and Use of Laboratory Animals, National Research Council, 2011.

### Virus isolation

Mosquitoes were captured at *Aotus* monkey-baited traps as part of an enzootic dengue study conducted in the vicinity of Iquitos, Peru [18]. Mosquitoes were identified to species, pooled (up to 25 specimens/pool), frozen on dry ice, and kept at -70°C until tested for infectious virus. Mosquito pools were triturated in 2 ml of diluent [10% heat-inactivated fetal bovine serum in Medium 199 with Earle's salts, NaHCO_3_ and penicillin (100 U/ml), streptomycin (100 μg/ml), and nystatin (100 U/ml)]. The suspensions were clarified by centrifugation (3,000 rpm for 10 min) and tested for virus by plaque assay on Vero (African green monkey kidney, ATCC CCL81) cell monolayers. A 0.l-ml aliquot of each original mosquito suspension and a 1:100 dilution of these suspensions were inoculated into duplicate wells of Vero cell monolayers. A second overlay, containing neutral red stain, was added 2 or 6 d later. If plaques were observed, the agar was removed, and the cells washed with fresh diluent and the resulting viral suspensions aliquoted into cryovials and frozen at –70^°^C. An aliquot of each suspension was inoculated onto confluent monolayers of Vero cells grown in a T-25 culture flask with 5 ml of liquid cell culture medium and observed daily for evidence of cytopathology. Cell cultures showing cytopathic effects were frozen at –70°C. Later, they were thawed, the suspension clarified by centrifugation at 3,000 rpm for 5 min, and then stored as 0.5-ml aliquots at –70°C for virus identification studies. The Vero passage 2 stock of one of these viruses, PE-M-0139 (isolated from a pool of 25 *Cx*. *portesi* mosquitoes captured in June 2002), was used in these studies.

### Sequencing

Total RNA from the Vero passage 2-cell culture supernatant was reverse transcribed using random hexamers, and the resulting cDNA was amplified using multiple displacement amplification. A sequencing library was prepared using the Nextera XT protocol, and sequenced on an Illumina HiSeq 2500 instrument. An initial HiSeq run of 47,871,860 reads was supplemented with a second HiSeq run of 204,323,558 reads, yielding 252,195,418 total 100bp paired-end reads (NCBI BioProject PRJNA290192).

### Metagenomic analysis

Initial analysis of the metagenomic sample involved a de novo assembly and taxonomic classification approach via MetAMOS [19], IDBA_UD [20], Kraken [21] and Krona [22]. However, initial inspection of the classified contigs and unassembled reads provided a convoluted picture of sample constituents, with only two reads classified as a member of the genus *Orthobunyavirus* (S1 Fig). LMAT [23](v1.2.3) was run on the dataset, only 5 reads were assigned to the genus *Orthobunyavirus*.

### Quality trimming and adapter removal

The reads were adapter clipped and quality trimmed using ea-utils, part of MetAMOS [19] (fastq-mcf command, default parameters) using the Nextera XT adapter sequence CTGTCTCTTATACACATCT.

### Targeted assembly and orthobunyavirus identification

To complement the *de novo* approach, putative orthobunyavirus reads were recruited to a diverse set of orthobunyavirus genomes via blastn [24](e-value 0.1, word size 7) using a custom orthobunyavirus database (Caraparu, Zungarococha, Oropouche viruses, containing L, M and S segments) downloaded from RefSeq [25]. The reference-based strategy filtered the nearly 50 million reads down to 234,280 paired-end reads (0.5% of the sample); blast did not report any read alignments to existing S segment sequences. Assembly of the recruited subset was performed with IDBA-UD (—pre_correction—num_threads 8—step 10); assembly was also attempted with SOAPdenovo [26] and Velvet-SC [27], but these produced fragmented assemblies.

The assembly was inspected for misassemblies by mapping all recruited reads back to the assembled contigs using Bowtie 2 [28]; a total of 121,901 reads mapped to the L segment (1762X avg. coverage) and 29,599 reads mapped to the M segment (617X avg. coverage). Coverage plots of the read mappings were visualized in IGV [29]. One junction in the assembled M segment was found to lack read support and was not consistent with related M segments (S4 Fig red arrow). A second round of recruitment was performed, including reads from the full assembly covering the region containing the erroneous deletion. The misassembled region was corrected after including these additional reads and rerunning IDBA_UD, resulting in consistent read support across both L and M segments.

### Full dataset de novo assembly

In addition, a full de novo assembly of the 50 million reads was performed (IDBA-UD [20], default parameters), resulting in 340,327 total contigs. Contigs assembled with the full HiSeq dataset were screened against the Human genome (hg19) and Green Monkey (BioProject PRJNA215854) draft sequence to identify host sequence and misassembled contigs. The recruited assembly was compared to the IDBA-UD [20] assembly on the full dataset using NUCmer [30].

### Sequence classification of S, M, and L segments

Orthobunyavirus (L and M) contigs were identified using both blastx and HMMER. An exhaustive search for the 900-1000bp S segment was performed, without success, using HMMER [31] (HMM profile http://pfam.xfam.org/family/PF00952, against all contigs using hmmpress and hmmscan).

#### Detection of S segment

As we failed to detect the S segment in the initial HiSeq run, we performed an additional sequencing run to facilitate the detection of the S segment of this novel Orthobunyavirus isolated from a pool of mosquitoes captured near Iquitos, Peru. The additional run provided over 200 million 100bp reads. All 200 million reads were first assembled with SOAPdenovo2 [32], and HMMER [31] (hmmsearch–E 1000 –cpu 4 HMM pfamseq) was used to align the 8,359,463 assembled contigs (translated to all 6-frames) against the nucleocaspid and non-structural protein HMMs. A single 608bp contig was shown to have significant hit (blastx, e-value = 2e-121) to Caraparu FMD0783 nucleocaspid protein (AGW82160.1), aligning at 89% aa identity across its entire length.

### Detection of terminal hairpin sequences

Based on known conserved terminal hairpin sequences found in the UTR regions in Orthobunyavirus genomes [33, 34], we searched for terminal hairpin sequences (AGTAGTGTGCT) near both 5’and 3’ends in the L, M, and S segments (within the first 300 nt) using BLAST [24] (e-value = 10, word size = 7), to determine if the assembly was complete on both ends.

### Sequence alignment and phylogenetic reconstruction

Amino acid (aa) sequences were aligned using MUSCLE [35] (default parameters), back translated to the original nucleotide sequences, edited to trim sequences from both ends that could not be reliably aligned, and then realigned with MUSCLE. Phylogenetic trees were subsequently reconstructed for both a global set of 101 orthobunyavirus genomes (L segment) and also on six Group C orthobunyavirus genomes (L and M segments), with FastTree2 [36]. Default parameters were used, and bootstrap support was determined by resampling the site likelihoods 1000 times and applying Shimodaira-Hasegawa test [37].

### Ability of El Huayo virus to replicate in a vertebrate host

To determine the potential for El Huayo virus to replicate in a vertebrate host, we inoculated three Syrian hamsters intraperitoneally with 0.2 ml of a suspension containing 10^6.5^ PFU/ml (10^5.8^ PFU/hamster) of the Vero passage 2 stock of El Huayo virus. The hamsters were anesthetized daily and three mosquitoes were allowed to take a blood meal from each of the hamsters. These engorged mosquitoes were then triturated individually in 1 ml of diluent and tested for infectious virus by plaque assay on Vero cells as described above. Hamsters were observed for 21 days for signs of illness.

## Results

### El Huayo virus sequencing

The El Huayo assembly yielded three contigs (Table 1) with alignments to orthobunyavirus sequences, with best hits for all three segments to Peruvian Caraparu strains [38]**.** We were unable to identify the known terminal hairpin sequences in the UTR regions, suggesting incomplete assembly of the segments and/or increased divergence in the known conserved region. The *de novo* assembly of the L and M segments with the first HiSeq dataset was more fragmented than the recruitment approach (95 contigs vs. 2 contigs) with >95% of aligned *de novo* contigs identical to the recruited assembly. However, the recruitment approach significantly reduced depth of coverage (50-fold average reduction in coverage for both segments), with a more dramatic effect on the M segment (100-fold) compared to L segment (5-fold), due to the high level of divergence from the reference strain. Differences between the two assemblies were investigated further with dnadiff [39]; the full de novo assembly had multiple small insertions with respect to the reference-recruited assembly. These insertions were found to have high identity hits to *Rhesus macaque* and Green monkey genomes, yet were lacking from both Caraparu genomes and the reference-recruited assembly. Closer inspection of these insertions identified them as retroviral sequences and contained within likely misassembled contigs (S3 Fig).

**Table 1:** Assembly statistics. ‘all’ indicates assembly generated from the full HiSeq dataset; ‘rec’ indicates assembly generated from the recruited subset; Cov indicates the combined coverage of each segment for both HiSeq runs.

Segment	# Contigs (all)	# Contigs (rec)	Cov	Length (nt)
**L**	65	1	1792X	6,746 bp
**M**	30	1	667X	4,721 bp
**S**	1	NA	25X	608 bp

**Fig 1:**
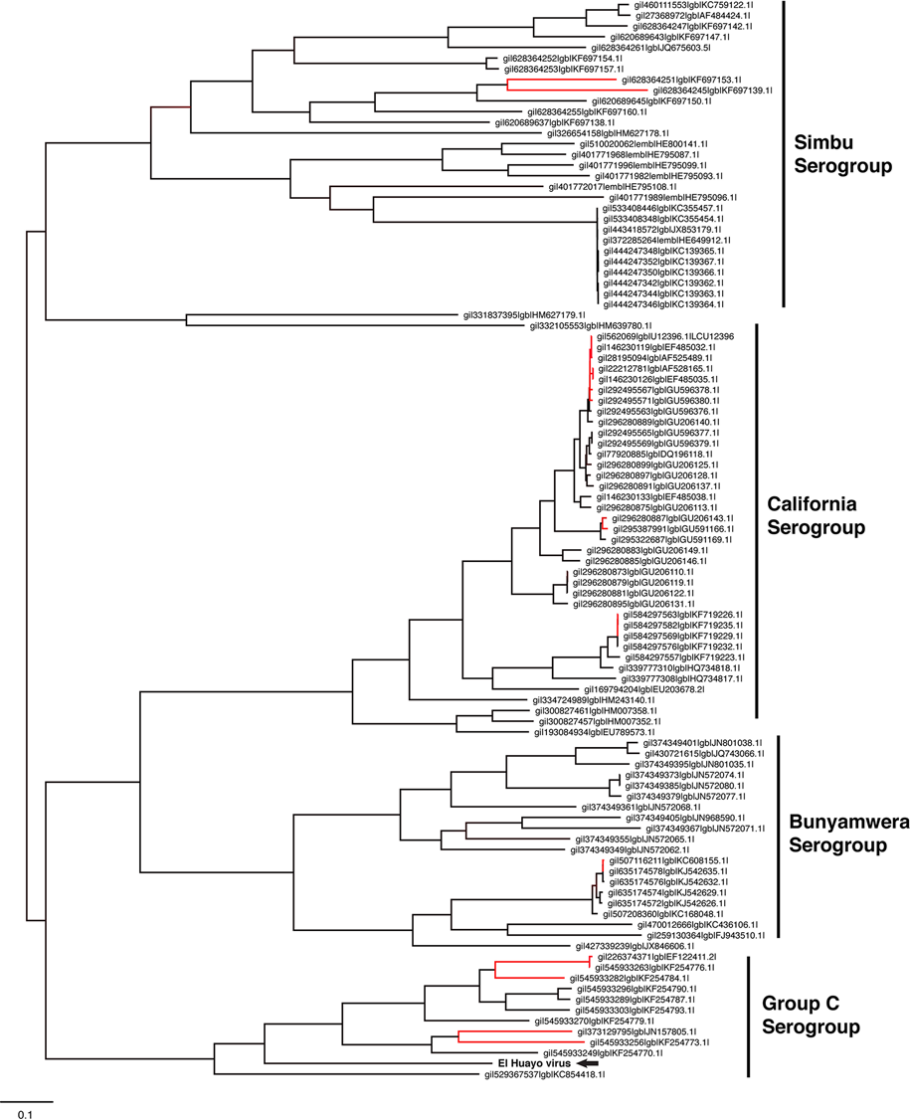
Maximum-likelihood phylogenetic placement (FastTree2) of El Huayo virus (Segment L). Strains colored in blue represent Group C orthobunyavirus genomes. Nodes with low bootstrap support (less than 0.8, Shimodaira- Hasegawa) are colored red. The strain in bold and indicated by the arrow indicates El Huayo virus, the novel strain sequenced in this study.

Phylogenetic analysis of the L segment suggests that this virus is closely related to Caraparu viruses comprising Group C orthobunyaviruses (Fig 1). We placed it within the Group C phylogeny, consistent with previously published phylogenetic relationships of orthobunyaviruses isolated from Peru [1]. El Huayo virus therefore appears to represent a novel, previously uncharacterized subclade of Group C viruses.

**Fig 2:**
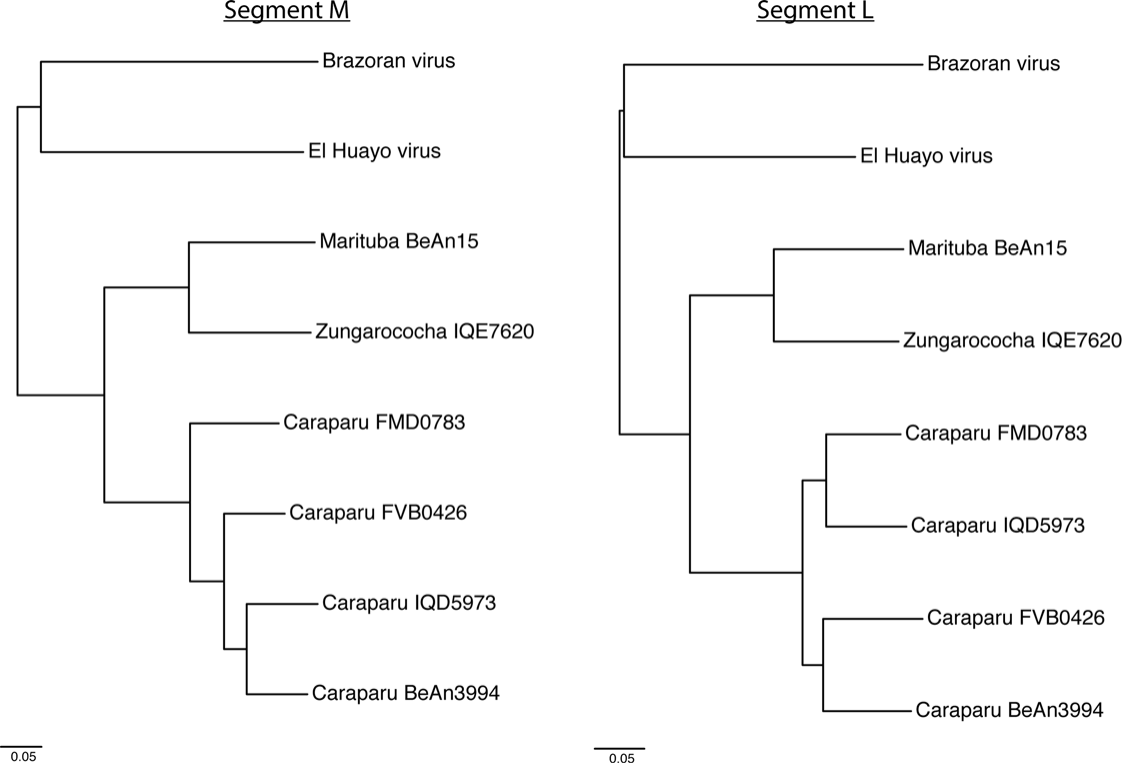
Group C phylogeny of orthobunyaviruses listed in Table 2, for both segment M and L. S segment not shown due to partial assembly (608 nt out of 1000–1100 nt). Nodes with low bootstrap support (less than 0.8, Shimodaira- Hasegawa) are colored red.

Orthobunyaviruses are known to have high rates of reassortment [40], and although both L and M segments were most closely related to Caraparu virus (Fig 2, Table 2), there is increased polymorphism observed in M relative to L compared to other orthobunyavirus genomes. In addition, Caraparu virus strain FMD0783 was found to be the most similar (nt/aa) to both the M and S segments, while strain IQD5973 (from Iquitos, Peru) was the most similar (nt/aa) to segment L.

**Table 2:** Nucleotide and amino acid similarity to the most closely related Group C orthobunyavirus genomes. Values in parenthesis indicate % identity calculated on segment M aligned regions without the highly polymorphic region located between positions 1500–2500.

Segment	Virus	Description	Location	Length (nt)	nt	aa
L	Brazoran	Houston	USA	6911 bp	64%	81%
**L**	**Caraparu**	**IQD5973**	**Peru**	**6794 bp**	**70%**	**84%**
****L****	**Caraparu**	**BeAn3994**	**Brazil**	**6855 bp**	**69%**	**84%**
L	Caraparu	FVB0426	Bolivia	6850 bp	69%	83%
**L**	**Caraparu**	**FMD0783**	**Peru**	**6849 bp**	**69%**	**84%**
L	Marituba	BeAn15	Brazil	6894 bp	69%	83%
L	Zungarococha	IQE7620	Peru	6936 bp	68%	83%
M	Brazoran	Houston	USA	4659 bp	62%	49% (70%)
**M**	**Caraparu**	**IQD5973**	**Peru**	**4290 bp**	**64%**	**53% (73%)**
M	Caraparu	BeAn3994	Brazil	4290 bp	63%	53% (70%)
M	Caraparu	FVB0426	Bolivia	4352 bp	63%	53% (72%)
**M**	**Caraparu**	**FMD0783**	**Peru**	**4349 bp**	**64%**	**53% (74%)**
M	Marituba	BeAn15	Brazil	4305 bp	62%	51% (71%)
M	Zungarococha	IQE7620	Peru	4538 bp	62%	52% (71%)
S	Brazoran	Houston	USA	1672 bp	—	—
S	Caraparu	IQD5973	Peru	1090 bp	80%	87%
**S**	**Caraparu**	**BeAn3994**	**Brazil**	**1048 bp**	**82%**	**89%**
S	Caraparu	FVB0426	Bolivia	1102 bp	81%	87%
**S**	**Caraparu**	**FMD0783**	**Peru**	**1068 bp**	**82%**	**89%**

### Ability of El Huayo virus to replicate in a vertebrate host

**Fig 3:**
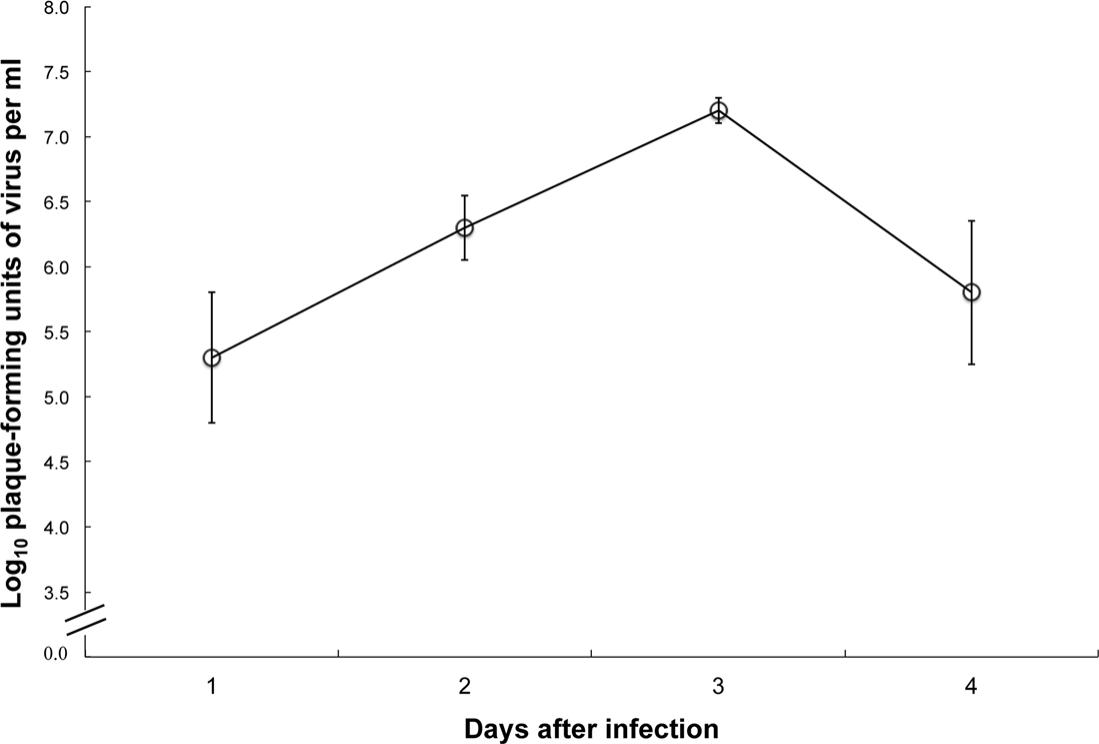
Viremia titers in Syrian hamsters by day after infection with El Huayo virus. Error bars indicate standard error.

El Huayo virus replicated to moderate titers in Syrian hamsters, with peak viremias of about 10^7.2^ PFU/ml occurring on day 3 after infection (Fig 3, Table 3). None of the hamsters displayed signs of illness, and all were well 21 days after infection.

**Table 3:** Replication of El Huayo virus in Syrian hamsters.

	Hamster number			
Day after infection	1	2	3	Mean (Std. Dev.)
1	5.8*	5.7	3.8	5.3 (1.0)
2	6.5	6.4	6.0	6.3 (0.5)
3	7.3	7.2	7.1	7.2 (0.2)
4	5.9	4.7	7.0	5.8 (1.1)

*Log_10_ plaque-forming units of virus per ml of blood

## Discussion

This is the first report of El Huayo virus, a novel member of the Group C orthobunyaviruses. Although rarely associated with human disease in nature, Group C viruses are known to cause febrile illness [13, 41]. The lack of reported cases is almost certainly due to a lack of diagnostic assays available for this group, and members of this group may be responsible for much of the dengue-like illnesses reported in areas of South and Central America where *Aedes aegypti* are not common [42]. In fact, Forshey et al. [17] estimated that about 2.5% of febrile illnesses in the region were due to infection with an orthobunyavirus, but were misdiagnosed as dengue.

*Culex portesi*, the species from which El Huayo virus was isolated, is a common species known to preferentially feed on rodents and marsupials [43, 44] and numerous viruses, including Caraparu-like viruses have been isolated from this species [45–47]. The ability of El Huayo virus to replicate to fairly high titer in hamsters indicates that like many other Group C virus, rodents may be involved in the natural maintenance cycle for this virus [48]. Thus, the natural cycle for El Huayo virus appears to be between *Cx*. *portesi* and rodents in the Amazon Basin region.

Because these viruses have a segmented genome, and because genetic reassortment has been demonstrated in this family/genus [49], the orthobunyaviruses are an ideal model for studying the evolution of novel viruses by genetic reassortment. How reassortment affects disease in humans and the ability of these viruses to replicate in vector species are key open questions.

In our initial comparative analysis, the best matches in our reference database shared ~60–80% nucleotide identity and 70–90% identity at the amino acid level with the (translated) novel S, M and L segment sequences, respectively. Given the low sequence identity of segment M relative to segment L, segment M might represent a novel reassortment; the region from 1500-2500bp contains a dramatic reduction in similarity to all known segment M strains available in RefSeq.

High divergence relative to existing genomes is a challenge for homology detection methods; sensitivity must be increased to detect divergent matches, but the increase in sensitivity also leads to potential misclassifications. Sensitive profile alignment methods based on hidden Markov models can detect protein domain signatures in cases where extreme divergence makes other methods infeasible [18], such as in the case of the highly divergent S segment recently reported for Brazoran virus [26] which was double the size of previously published orthobunyavirus S segments. Its S segment contained no known homology to existing segment S proteins; however, similar to what we report here, it did have conserved orthobunyavirus domains that were detected via InterProScan [27]. While insufficient sequencing depth in our initial HiSeq run prohibited detection of the S segment, adding another HiSeq run allowed for the detection of this small viral segment. This result highlights that lower abundance sequences in environment samples may often be missed, and sequencing depth is still an important tool for uncovering low abundance novel viruses from metagenomic samples.

Based on amino acid sequence similarity, the orthobunyavirus genome of El Huayo virus reported in this study is most closely related to Caraparu virus Peruvian strains IQD5973 and FMD0783 [38], both recently deposited in GenBank. This recent growth in publicly available Group C orthobunyavirus genomes enabled the reliable placement of our novel strain within the Group C serogroup. Prior to Huang et al. 2014, there were no complete genomes (including all three segments) from within Group C. Lack of complete genomic sequences of serogroups of interest can lead to misclassification or misidentification, evidenced by a recent study that reported that a collection of Group C genomes likely require further validation [38]. This highlights the importance of efforts to populate reference databases. There exists a vast underrepresentation of viral diversity for various clades, and of particular interest to this study, there are only a small number of South American orthobunyavirus sequences. Continuing efforts are required to fill out viral reference databases to ensure reliable identification and characterization of novel *Bunyaviridae* genomes.

An additional confounding factor for novel virus identification and assembly is host endogenous retroviral elements [50] (S2 Fig and S3 Fig). Aggressive assembly strategies can result in chimeric host-plus-virus assemblies in which sequence shared by both virus and host results in false joins between the two genomes; specifically, retroviral elements integrated into host genomes. We have shown that a recruitment-based strategy, even at relatively high levels of amino acid divergence, can prove useful for avoiding co-assembly of host and target virus. However, this approach requires the presence of reference strains in the database and is prone to under-recruitment of reads in highly polymorphic regions. In summary, while advances in sequencing technology allow for the discovery of novel viruses present at low abundances in a sample, care must be taken to properly address confounding factors.

## Supporting Information

S1 Fig **A.** Krona visualization of Kraken-based classification of entire sample. *Chlorocebus aethiops* (Green Monkey) is the host sequence used for cell cultures; Unknown indicates 77% of the reads were unable to be classified. There were 4770 total reads (0.01%) classified as Viruses (including RNA Viruses). **B.** Krona visualization of Kraken-based classification of putative viral reads. Of the 4770 viral reads, only 0.03% were assigned to the genus *Orthobunyavirus*, representing two reads out of the nearly 50 million total (0.000003%).
(PNG)

S2 Fig Illustration of an identified segment L misassembly. Assembly on the full dataset resulted in a handful of misassembled contigs that incorrectly joined orthobunyavirus segment L with host retrovirus elements. The horizontal black line at the top represents a region from the Green Monkey genome, the red line indicates a shared k-mer (20) between Segment L and the endogenous retroviral element, the green line represents the retrovirus and blue lines represent segment L.
(PNG)

S3 Fig IGV plot showing a misassembled contig produced by the full de novo assembly. The read pileup on the right hand side of the figure corresponds to a high coverage assembly of a small region of segment L, while the read pileup at reduced coverage found on the left-hand side corresponds to Green Monkey chromosome 9 (aligns across entire length at 99% identity).
(PNG)

S4 Fig IGV plot of the read pileup of segment M reference-recruited assembly. The red arrow indicates the false join at positions 995-1005bp in the assembly, lacking clear read support.
(PNG)
