# Supplementary figures and images for: Identification and Genomic Analysis of a Novel Group C Orthobunyavirus Isolated from a Mosquito Captured near Iquitos, Peru
Source: PLoS Negl Trop Dis. 2016 Apr 13;10(4):e0004440. doi: 10.1371/journal.pntd.0004440 (PMC4830577; doi:10.1371/journal.pntd.0004440)

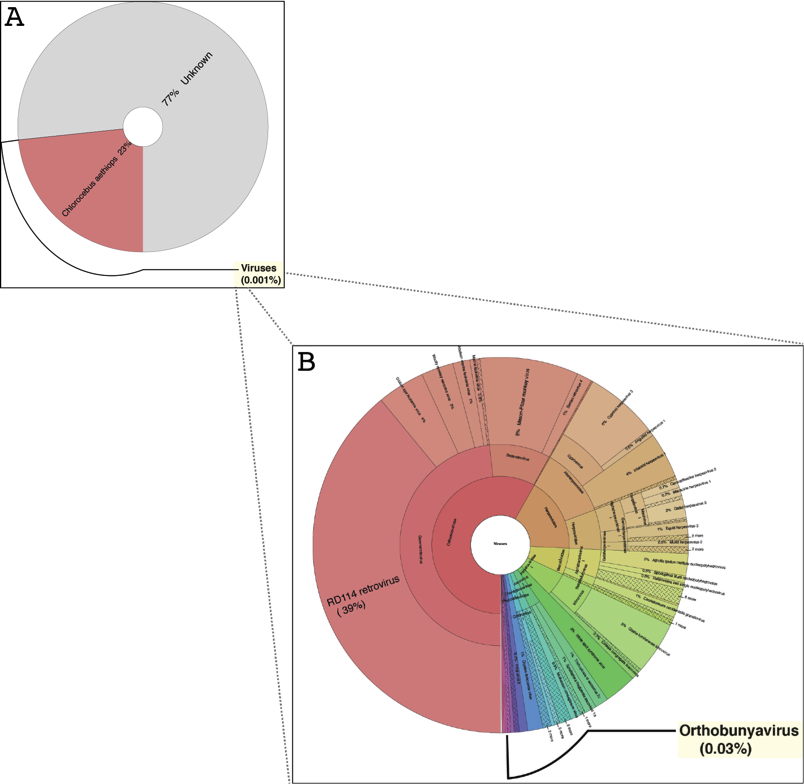

Supplement: S1 Fig — A. Krona visualization of Kraken-based classification of entire sample. Chlorocebus aethiops (Green Monkey) is the host sequence used for cell cultures; Unknown indicates 77% of the reads were unable to be classified. There were 4770 total reads (0.01%) classified as Viruses (including RNA Viruses). B. Krona visualization of Kraken-based classification of putative viral reads. Of the 4770 viral reads, only 0.03% were assigned to the genus Orthobunyavirus, representing two reads out of the nearly 50 million total (0.000003%). (PNG) [file pntd.0004440.s001.png]

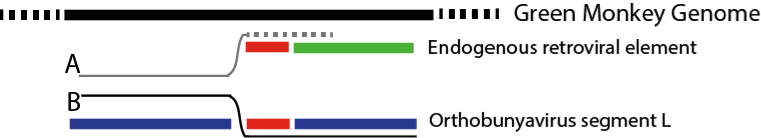

Supplement: S2 Fig — Assembly on the full dataset resulted in a handful of misassembled contigs that incorrectly joined orthobunyavirus segment L with host retrovirus elements. The horizontal black line at the top represents a region from the Green Monkey genome, the red line indicates a shared k-mer (20) between Segment L and the endogenous retroviral element, the green line represents the retrovirus and blue lines represent segment L. (PNG) [file pntd.0004440.s002.png]

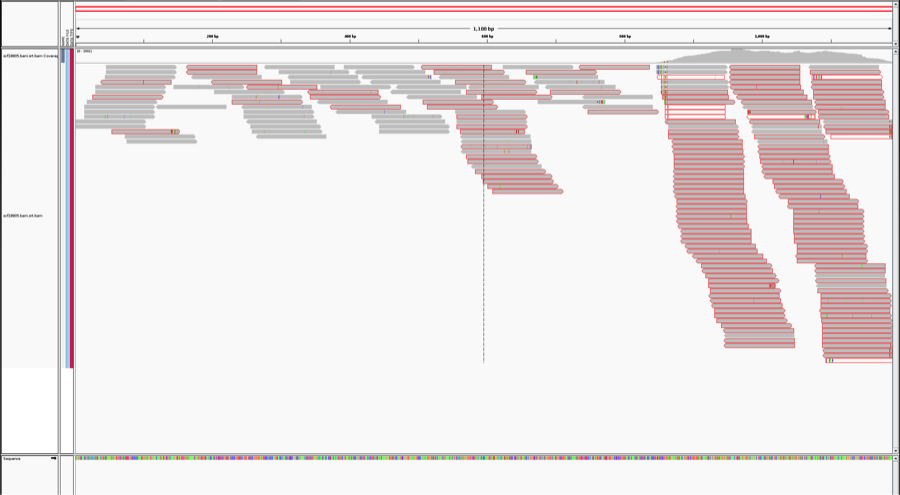

Supplement: S3 Fig — The read pileup on the right hand side of the figure corresponds to a high coverage assembly of a small region of segment L, while the read pileup at reduced coverage found on the left-hand side corresponds to Green Monkey chromosome 9 (aligns across entire length at 99% identity). (PNG) [file pntd.0004440.s003.png]

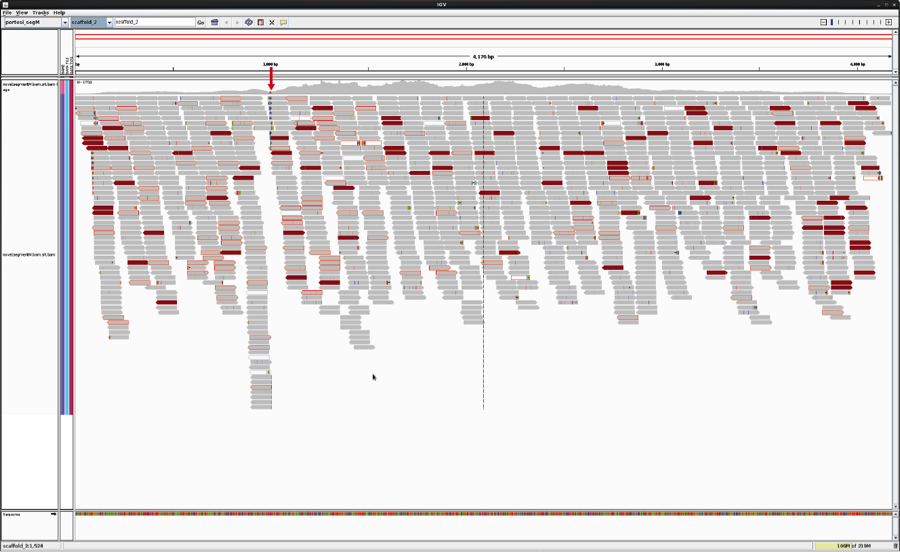

Supplement: S4 Fig — The red arrow indicates the false join at positions 995-1005bp in the assembly, lacking clear read support. (PNG) [file pntd.0004440.s004.png]
